# The Role of Circular RNA in the Progression of Gliomas and Its Potential Clinical Applications

**DOI:** 10.3390/biology14070795

**Published:** 2025-06-30

**Authors:** Wen Wu, Menglei Xiong, Chen Jiang, Xinru Zhou, Yingjie Ma, Tao Wang, Shan He, Baicheng Ma

**Affiliations:** Jiangxi Provincial Key Laboratory of Cell Precision Therapy, School of Basic Medical Sciences, Jiujiang University, Jiujiang 332005, China; 20210207123@jju.edu.cn (W.W.); 13697981610@163.com (M.X.); 20210204374@jju.edu.cn (C.J.); 20210203154@jju.edu.cn (X.Z.); xmxm0611@163.com (Y.M.); comwangtaocom@163.com (T.W.)

**Keywords:** circRNAs, glioma, RNA-binding protein (RBP), MiRNA sponge, blood–brain barrier (BBB), clinical therapy

## Abstract

Circular RNAs (circRNAs) are single-stranded noncoding RNAs with a covalently closed loop structure. They are known for their stability, abundance, and highly conserved nature. Their expression is often specific to tissues or developmental stages. They interact with microRNAs (miRNAs) and RNA-binding proteins (RBPs) and they undergo N^6^-methyladenosine (m^6^A) modifications, further affecting gene transcription and translation. Increasing evidence over the past decades has revealed that dysregulated circRNA expression is associated with various neurological disorders, particularly the glioma, one of the most malignant tumors with a poor prognosis. Due to the presence of the blood–brain barrier (BBB) and drug resistance, conventional therapeutic approaches have shown limited efficacy. Recently, increasing attention has been directed toward precisely targeted therapies, with circRNAs emerging as promising molecules for cancer treatment. Studies indicate that circRNAs play a key role in glioma proliferation and metastasis. Substantial evidence indicates that exosomes can package circRNAs and facilitate their transport across the BBB into brain tissue, highlighting the potential of circRNAs as therapeutic targets for glioma. This review summarizes circRNAs’ functional mechanisms, clinical application relevance, and current limitations. It offers future research directions in this evolving field, aiming to encourage further research on circRNAs’ therapeutic applications and contribute to the development of novel glioma-treatment strategies.

## Simple Summary

The treatment outcomes and prognosis for glioma patients remain unsatisfactory, and a comprehensive understanding of the mechanisms underlying glioma development and progression is essential for improving therapeutic strategies. It has been demonstrated that the etiology of gliomas involves differential expression of various circular RNAs (circRNAs). This review outlines the intrinsic biological mechanisms of circRNAs, then presents many circRNAs that contribute to tumor progression through different pathways, emphasizing their potential as biomarkers. Moreover, this review explores the correlations among circRNAs, gliomas, and clinical drug responses, integrating clinical challenges to highlight the diagnostic, prognostic, and therapeutic relevance of circRNAs. Therefore, this review emphasizes that circRNAs, due to their unique advantages and high structural stability, may serve as promising targets for future glioma treatment by blocking drug-resistance transmission among tumor cells, restoring drug sensitivity, encoding functional proteins for immunotherapy, and regulating blood–brain barrier permeability to therapeutic agents.

## 1. Introduction

Over the past decades, extensive experimental studies have progressively revealed the fundamental structure and characteristics of circRNAs. Unlike linear RNAs with 5′ caps and 3′ tails, circRNAs are defined by their covalently closed loop structure without 5′ caps and 3′ tails. These circRNAs are formed by back-splicing precursor mRNA (pre-mRNA) exons [1]. They are a class of endogenous noncoding single-stranded RNAs characterized by a closed-loop structure, great stability, abundance, conservation, and tissue and developmental stage-associated expression. Based on their biogenesis from different genomic regions, circular RNAs can be categorized into four types: (i) exon-intron circRNAs (eIciRNAs) [2], (ii) exonic circRNAs (ecircRNAs) [3], (iii) circular intronic RNA (ciRNAs) [4], and (iv) tRNA intronic circRNAs (tricRNAs) [5]. The distinct distribution patterns of these circRNAs contribute to their diverse functional mechanisms. However, recent research has indicated that circRNAs are no longer strictly “noncoding” RNAs, as m^6^A modifications can enable their translation into proteins [2]. In recent years, the signaling pathways underlying the role of circRNAs in various cancers have been gradually discovered and increasingly understood. Previous research has demonstrated a strong association between circRNAs and the occurrence and development of gliomas. CircRNAs affect glioma pathophysiology through multiple mechanisms involving primarily RNA-binding proteins (RBPs), miRNA sponging, and exosomal packaging, collectively forming a complex regulatory network (Supplementary Table S1). Understanding existing regulatory mechanisms and identifying novel circRNA-associated pathways may provide significant insights into overcoming gliomas.

Gliomas are the most common primary brain tumors in adults [6] and are characterized by various genetic molecular aberrations [7]. These tumors originate from neuroglial progenitor cells and show high invasiveness. Gliomas have been classified into five categories based on histopathological criteria: astrocytic tumors, oligodendrogliomas, oligoastrocytomas, ependymal, and neuronal or mixed neuronal-glial tumors (i.e., gangliogliomas) [8]. The annual incidence rate of central nervous system neoplasms is 3.9/100,000 [9], of which gliomas account for more than 40% [10], and managing gliomas remains challenging due to their invasive nature and frequent recurrence [9,11]. Despite the use of chemotherapy, radiotherapy, and immunotherapy [5] to manage glioma invasion, patients often develop resistance to these treatments as the tumor progresses. Gliomas are prone to relapse, with increased resistance to therapy observed upon recurrence. Research has identified four characteristic molecular subtypes of gliomas—classical (CL), neural (NE), mesenchymal (MES), and proneural (PN), with each cluster closely associated with specific genomic abnormalities in glioma cells [12]. Moreover, experimental studies have shown that the transition from PN to MES subtypes during disease recurrence is associated with increased treatment resistance in glioblastoma (GBM) relapse [13]. Thus, the overall cure rate for GBM remains low, and the prognosis is unfavorable. Data reveal that the median overall survival is <20 months, with a 5-year survival rate of <3% [14].

Current treatment strategies for gliomas have yielded limited therapeutic success. A comprehensive review of the literature suggests that this is primarily due to the presence of the blood–brain barrier (BBB) and the blood–tumor barrier (BTB) [15], as well as the progressive development of drug resistance. Various studies have established that circRNAs play pivotal roles in the pathophysiological processes of gliomas. Recent advances in drug development have demonstrated that integrating circRNAs with delivery platforms, such as manganese-based nanoenzymes [16], exosomes [17], and lipid nanoparticles [18], can facilitate efficient penetration of the BBB. At the same time, the combination of circRNAs with epidermal growth factor receptor (EGFR) [19] or histone deacetylase (HDAC) inhibitors [20] has shown significant potential in restoring drug sensitivity in resistant glioma cells. Moreover, circRNAs possessing a protein-coding capacity have emerged as promising candidates for developing circRNA-based vaccines. In summary, circRNAs can serve as both potential predictive biomarkers and promising therapeutic targets for gliomas.

## 2. CircRNAs Interact with RBPs

**Figure 1:**
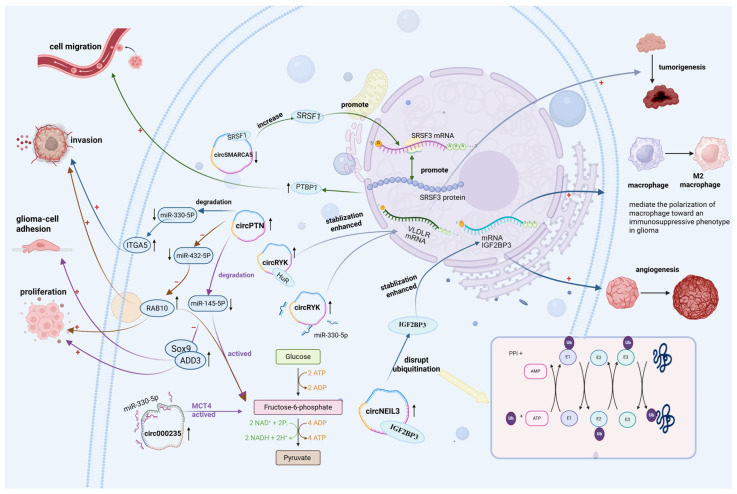
CircRNAs act as miRNA sponges and bind to RBPs. CircSMARCA5 acts as a glioma suppressor by binding to and reducing the levels of the RBP SRSF1. However, the original downregulated level of circSMARCA5 can increase SRSF1 in glioma, subsequently promoting SRSF3 mRNA transcription and SRSF3 protein translation, which increase PTBP1 transcriptor level. CircRYK increases the expression and stability of the oncogene *VLDLR* mRNA by associating with the RBP HuR and sponging miR-330-5p, subsequently propelling tumorigenesis and development of glioma. CircNEIL3 plays a negative role in gliomas, with a high expressed level, not only promoting its macrophages’ immunosuppressive properties by facilitating macrophage polarization to M2 type but also increasing its expression by interacting with IGF2BP3 and inhibiting protein ubiquitination, thereby accelerating its angiogenesis. Circ000235 and circPTN both activate glioma-cell glycolysis by sponging miR-330-5p and miR-145-5p, respectively. Nonetheless, circPTN facilitates glioma proliferation, adhesion, and invasion by sponging miR-145-5p, which suppresses the activity of oncogenic proteins Sox9 and cell adhesion-associated molecule adducin 3 (ADD3). On the other hand, circ-PTN can directly sponge miR-432-5p and target RAB10 protein to accelerate tumor invasion and glycolysis. +, it represents the meaning of promotion; −, it represents the meaning of inhibition or reduction; ↑, it represents the meaning of elevation; ↓, it represents the meaning of decrease.

CircRNAs influence the concentration and stability of RBPs or their target genes through direct interactions (Figure 1). A study showed that has-circ-0001445 (circSMARCA5) substantially decreased in the glioma tissue and indirectly regulates the expression of serine/arginine-rich splicing factor 3 (SRSF3) isoforms by interacting with serine and arginine-rich splicing factor 1(SRSF1). As a well-characterized oncoprotein, SRSF1 promotes glioma-cell migration. The findings suggest a negative correlation between circSMARCA5 expression and glioma progression [21], highlighting its potential role as a tumor suppressor through RBP SRSF1 sequestration. Another study indicated that circNEIL3 is upregulated in glioma tissues and coordinates with IGF2BP3, which not only promotes its immunosuppressive properties but also elevates its expression level [22]. At the same time, IGF2BP3 is protected from ubiquitination, maintaining its stability [23]. Furthermore, it has been found that circRYK expression was increased in gliomas, which always symbolizes a poor prognosis. Mechanistically, circRYK drives and accelerates the expression of oncogene VLDLR via sponging miR-330-5p [24] and is also capable of maintaining the stability of mRNA VLDLR by forming an association with the RBP HuR. In a nutshell, circRYK promotes tumorigenesis and metastasis through interactions with both RBPs and miRNAs in glioma tissues. In the previous evaluations, it is evident that a few circRNAs and RBPs were closely associated with gliomas. Finally, these findings indicate that multiple circRNAs and RBPs are closely associated with gliomas, and any dysregulation within the circRNA–RBP axis may contribute to glioma pathogenesis by promoting tumor proliferation and invasion.

## 3. CircRNAs Act as miRNA Sponges

Research shows that circRNAs are mainly located in the cytoplasm, where they function as miRNA sponges by harboring microRNA response elements (MREs). Generally, a single circRNA contains multiple distinct miRNA binding sites, which allows different miRNAs to bind to these circRNAs (Figure 1). A recent study demonstrated that circPTN, which is highly expressed in gliomas, induces cell proliferation, invasion, and glycolysis by sponging miR-432-3p in gliomas [25]. These findings suggest that circPTN may serve as a biomarker for monitoring cisplatin efficacy in GBM patients. Moreover, another study indicated that circPTN facilitates frizzled-4 expression and activates the Wnt/β-catenin pathway by sponging miR-145-5p, contributing to the pathogenesis of rheumatoid arthritis. Multiple circRNAs can sponge a single miRNA. A recent study has shown that the upregulation of circRYK can accelerate glioma tumorigenesis by sponging miR-330-5p [24]. Similarly, circ000235 is highly expressed in bladder cancer and is associated with poor prognosis by promoting glycolysis via the activation of monocarboxylate transporter 4 through miR-330-5p sponging. Moreover, circPTN also exerts a significant effect on glioma progression by targeting miR-330-5p [26]. These findings highlight the complexity of disease progression, which often involves a network of regulatory interactions rather than a single factor. As research continues to expand, the interplay between circRNAs and miRNAs may provide promising therapeutic targets for gliomas and other diseases.

## 4. CircRNAs Regulate Transcription and Affect Their Parental Genes

**Figure 2:**
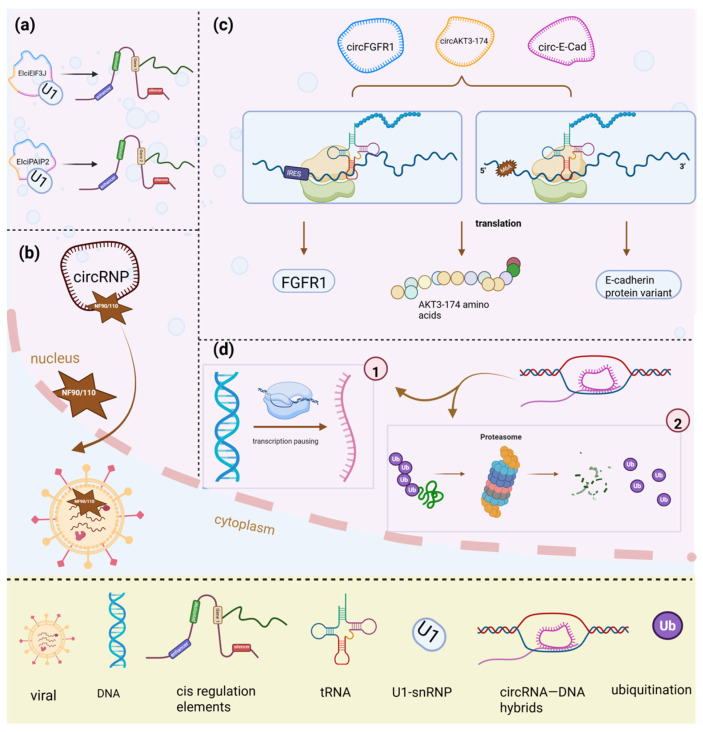
The other mechanism of circRNAs in physiological and pathological conditions. (**a**) EIciEIF3J and EIciPAIP2 promote EIF3J and PAIP2 gene transcription by recruiting U1 snRNP in *cis*. (**b**) CircRNP, composed of NF90/NF110, releases NF90/NF110 upon stimulation, allowing its binding to viral mRNA and promoting antiviral immunity. (**c**) CircFGFR1, circAKT3-174, and circ-E-cad encode proteins FGFR1, AKT3-174, and C-E-cad, respectively, through IRES- and m^6^A-mediated cap-independent translation. (**d**) CircSMARCA5 interacts with its parental gene locus, forming an R-loop contributing to transcriptional pausing and proteasome inhibition; IRES, internal ribosome entry site; m^6^A, N6-methyladenosine; U1-snRNP, U1 small nuclear ribonucleoprotein.

Evidence suggests that nuclear-retained circRNAs cannot only activate transcriptional expression but also regulate their parental genes. For instance, eIciRNAs are a subclassification of circRNAs primarily located in the nucleus and can bind with U1 snRNPs, thus positively regulating the transcription of their parental genes in cis [2]. EIciEIF3J and EIciPAIP2 promote the transcription of EIF3J and PAIP2 genes by U1 snRNPs [27] (Figure 2a). Further, the CiRNAs are limited to introns and have the potential to stimulate RNA polymerase-II transcription in the nucleus to affect its parental genes [4].

CircRNAs regulate the transcription of their parental genes and can also directly bind to DNA, forming RNA–DNA loops. CircRNA–DNA hybrids have been shown to promote transcriptional pausing, proteasome inhibition, chromatin reorganization, and DNA breakage in acute leukemia [28]. Furthermore, circSMARCA5 interacts with its parent gene locus, leading to the formation of an R-loop, which contributes to transcriptional pausing at exon 15 of *SMARCA5* [21] (Figure 2d). Similarly, studies have revealed that circSEP3 strongly binds to its cognate DNA, forming an RNA–DNA hybrid or R-loop, further contributing to transcriptional pausing [29].

## 5. Translation of circRNAs into Functional Proteins

CircRNAs not only regulate the transcription of their parental genes in *cis* or the promoter in cognate loci but can also undergo translation into functional proteins [30,31,32]. Recent research has revealed that certain cytoplasmic circRNAs can be efficiently translated into detectable peptides. The Internal Ribosome Entry site (IRES) (without a 5′ cap) and N^6^-methyladenosines (m^6^A)-mediated cap-independent translation would be the underlying mechanism for circRNAs translation [33] (Figure 2c).

A relevant study demonstrated that fibroblast growth factor receptor 1 (FGFR1) is encoded by circFGFR1, which is downregulated in cancer. Its translation depends on IRES activity, and the resulting circFGFR1-derived peptide (circFGFR1-p) has been found to suppress cell growth under stress conditions [34]. Similarly, the translation of circAKT3-174 leads to the production of AKT3-174 amino acids (aa) through IRES-mediated translation, and the overexpression of AKT3-174aa has been shown to reduce cell proliferation, radiation resistance, and glioma tumorigenesis [35]. Furthermore, a circular E-cadherin RNA (circ-E-Cad) encodes a secretory E-cadherin protein variant (C-E-Cad), which promotes GBM tumorigenesis by binding to the EGFR CR2 domain and maintaining glioma stem-cell tumorigenicity. Another study indicated that circZNF609 regulates myoblast propagation and encodes a protein in a splicing-dependent, cap-independent manner in Duchenne muscular dystrophy (DMD) myoblasts under the stress condition [36]. Research on m^6^A modification suggests that the depletion of YTHDF3 significantly inhibits GFP production from circRNAs, while the knockdown of eIF4G2 has an even more pronounced effect in reducing circRNA-derived protein translation [37]. Moreover, not only can circRNAs translate certain proteins, but they also play a role in interrupting mRNA to translate protein. The study has indicated that circYAP has been shown to inhibit tumor progression by disrupting the assembly of the YAP mRNA translation initiation complex through interactions with eIF4G and PABP, thereby interfering with protein synthesis [38]. While it is now established that circRNAs can be translated into proteins, the precise mechanisms and regulatory pathways driving this process remain incompletely understood [31].

## 6. Interaction of CircRNAs and Viral mRNA in a Pathological Environment

Beyond regulating parental gene transcription and undergoing protein translation under stress or physiological conditions, circRNAs also interact with viral mRNA in pathological environments. Li X et al. demonstrated that circRNA expression progressively decreases during viral infection. Their investigation revealed that under physiological conditions, circRNP complexes were composed of NF90/NF110 bound to the introns of circRNP in the nucleus, where NF90/NF110 can promote the production of circRNP and induce its translocation from the nucleus to the cytoplasm, thus stabilizing circRNP (Figure 2b). In viral infection, NF90/NF110 from circRNP complexes is released and binds with viral mRNA to improve antiviral immunity [39]. Another study showed that infection with Kaposi’s sarcoma-associated herpesvirus (KSHV) induces the production of various circRNAs, among which circ-0001400 exhibits the most significant upregulation. Although increased circ-0001400 levels do not prevent KSHV from infecting normal cells, they inhibit KSHV gene expression and promote TGF-α expression, contributing to antiviral immunity [40].

## 7. CircRNAs Translocate Proteins

Recent studies have revealed that circRNAs can regulate protein translocation and participate in the formation of circRNA-protein-mRNA ternary complexes to facilitate translation [41]. CircAmotl1 interacts with c-Myc, increasing its nuclear translocation, stabilizing its concentration, and increasing its affinity for target genes, ultimately promoting tumorigenesis [42]. Moreover, circRNAs can regulate protein translocation to the cytoplasm. In hepatocellular carcinoma, circFOXP1 enhances the Warburg effect by promoting the nuclear-to-cytoplasmic translocation of PTBP1 and increasing its expression [43]. Similarly, high circBACH1 expression is involved in hepatocellular carcinoma progression by facilitating the translocation of HuR from the nucleus to the cytoplasm [44]. Another mechanism involving circRNAs is their role in regulating translation initiation.

## 8. The Role of CircRNAs in Glioma Development and Invasion

**Figure 3:**
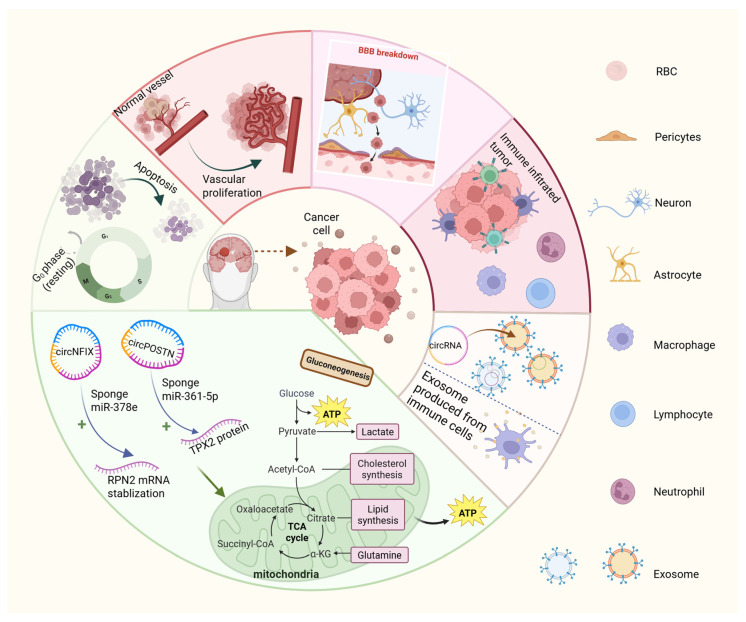
Six mechanisms by which circRNAs contribute to glioma tumorigenesis, proliferation, and invasion. These include altering glioma-cell glucose metabolism, promoting angiogenesis and tumor proliferation, disrupting normal cellular processes and apoptosis regulation, impairing the blood–brain barrier (BBB), modulating immune responses, and facilitating exosome-mediated circRNA transport. RBC, red blood cell. +, it represents the meaning of promotion.

Increasing research indicates that the dysregulation of circRNAs exerts both tumor-promoting and tumor-suppressive effects on glioma growth and progression. To facilitate the clinical application of circRNAs, it is imperative to conduct comprehensive studies on the association and mechanism of action between circRNAs and gliomas and to identify the fundamental interactions between various complex mechanisms (Supplementary Table S2). The primary pathways through which circRNAs affects gliomas are outlined below (Figure 3).

### 8.1. CircRNAs Promote Angiogenesis and Proliferation of Tumor Tissues

Blood vessels are the primary component of most cancer cells, as they directly supply oxygen and nutrition. Consequently, these vessels are also an efficient and convenient conduit for cancer cells to facilitate rapid proliferation and invasion and to disperse to other organs. Research has demonstrated that circNEIL3 overexpression substantially increases the protein expression of SPP1, which has an evident effect on stimulating angiogenesis in gliomas [22]. EWS RNA-binding protein 1 (EWSR1) increases the circularization of circNEIL3, leading to its upregulation and subsequent glioma progression. Similarly, another recent study identified that circPITX1 overexpression increases glioma-cell proliferation and migration by facilitating angiogenesis as well. Mechanistically, circPITX1 targets miR-584-5p, which is upregulated in glioma. Experimental findings have suggested that the upregulation of miR-584-5p reduces the effects of circPITX1 overexpression. Therefore, circPITX1 and miR-584-5p jointly regulate glioma progression by modulating growth, angiogenesis, and cellular infiltration [45].

CircRNAs also directly promote tumor-cell proliferation. A recent study identified that upregulated circTTBK2 can promote proliferation, migration, and invasion by sponging miR-217 in gliomas [46]. Further, miR-217 directly targets hepatocyte nuclear factor 1 beta (HNF1β), which contains a binding site for oncogenic protein Derlin-1 [47]. Another study revealed that circPTN, which is highly expressed in glioma tissues, facilitates tumor proliferation by sponging miR-145-5p and miR-330-5p [26]. These findings highlight the role of circRNAs in glioma pathogenesis and highlight their potential as therapeutic targets.

### 8.2. CircRNAs Destroy Normal Cellular Physiological Processes

CircPTN plays a dual role in glioma progression, contributing not only to tumor-cell proliferation but also to the self-renewal and development of glioma stem cells [26]. As a form of regulated cell death (RCD), apoptosis can maintain cellular homeostasis by stimulating the mechanism of cell suicide in mutated or damaged cells. Research has demonstrated that circRNAs regulate glioma apoptosis [48,49,50] and other normal physiological processes.

Beyond apoptosis, RCD also includes ferroptosis, necroptosis, pyroptosis, cuproptosis [48], and autophagy [49], which are associated with tumor-cell proliferation and metastasis. These processes are closely associated with both tumorigenesis and neurological disorders, and the therapeutic potential of ferroptosis induction in gliomas has been established [51]. As well as research establishing that circTTBK2 promotes proliferation and invasion by sponging miR-217, another piece of research has elucidated that it can also regulate ferroptosis by activating integrin subunit beta 8 (ITGB8) through the sponging of miR-761 [52]. These findings highlight the complex regulatory roles of circRNAs in glioma pathophysiology and their potential as therapeutic targets.

### 8.3. CircRNAs Disrupt Cellular Energetics of Gliomas

Cancer cells are different from normal cells subject to regulated cell death, which maintain rapid proliferation and division by reprogramming their glucose metabolism and energy-production pathways to support the requirement of high energy intake. Research has demonstrated that circRNAs are involved in regulating glycolysis [53]. Simultaneously, the researchers discovered that the level of circNFIX was increased in glioma tissue, which protected RPN2 mRNA from degradation in gliomas via sponging miR-378e, thus improving glucose metabolism in glioma cells [54]. Furthermore, the circPOSTN expression increased in glioma tissues, which in turn controls the expression of TPX2 via sponge miR-361-5p [55], ultimately promoting the growth and aerobic glycolysis of glioma tissues. Another study suggested that circSOBP expression levels were reduced in gliomas, which could suppress glycolysis through interaction with TKFC to disrupt cellular energetics and inhibit glioma progression [56]. CircRNAs offer novel therapeutic avenues for targeting glioma energy metabolism.

### 8.4. CircRNAs in the Blood–Brain Barrier (BBB) and the Blood–Tumor Barrier (BTB)

In addition to influencing tumor energy metabolism, circRNAs are also involved in regulating the permeability and integrity of the blood–brain barrier (BBB) [57]. The BBB is a critical component of the central nervous system (CNS) that provides regular physiological function and high stability of the CNS’s activities [58]. The absence of fenestrations in the BBB due to its continuous tight junctions substantially restricts the movement of neurotoxic molecules (viruses, inflammatory factors, and heavy metals) via the endothelial cell layer [59]. However, this also restricts the drug’s ability to reach the brain. Recent evidence indicates that gliomas not only facilitate BBB remodeling but also contribute to the formation of BTB [60]. Glioma endothelial cells (GECs) act as the main structural component of the BTB, which is highly heterogeneous and exhibits distinct features, including non-uniform permeability, active efflux of molecules, and restricted delivery of antitumor drugs to glioma tissues. Although the BTB is considered ‘leakier’ than the BBB [15], it retains key characteristics of the BBB, such as the expression of active efflux transporters in endothelial and tumor cells [61]. These findings suggest that the BTB represents a compromised form of the BBB [62].

Recent research has demonstrated that the circUSP1 involves preserving the integrity of the BBB and reducing its permeability. The knockdown experiment evidently observed that facilitating the permeation of the antitumor drug doxorubicin across the BTB into tumor tissue induces glioma-cell apoptosis. This process may contribute to the development of novel therapeutics for gliomas. CircUSP1 increases the expression of tight junction-associated proteins, including occludin, claudin-5, and ZO-1, by sequestering miR-194-5p in glioma endothelial cells (GECs) within the BTB [63]. Another study suggested that circ-001160 exerts antitumor effects by modulating BTB permeability through its interaction with miR-195-5p [64]. Wu P et al. clarified that KHDRBS3 (RNA binding protein) and circDENND4 were upregulated in GECs. Meanwhile, KHDRBS3 can increase circDENND4 stability, and CircDENND4 plays a negative role in BTB infiltration, which upregulates the expression of tight junction-related proteins via sponging with miR-577 [65]. CircRNAs are considered key regulators of BBB/BTB permeability by affecting the proliferation and migration of GECs [65].

### 8.5. CircRNAs Mediate Tumorigenesis Through Cytokines and Immune Cells

When it comes to defending disease, the immune system continues to be a subject worthy of in-depth study and research. Immunosuppressive factors and immune cells within the tumor immune microenvironment contribute to glioma progression, making therapies targeting the immune microenvironment a key research focus. Studies suggest that circSOBP interacts preferentially with the C-terminus of the TKFC protein, promoting glioma-cell proliferation and migration by activating the IKKε/TBK1/IRF3 signaling pathway mediated by MDA5 [56]. Encouragingly, the activation of the MDA5 pathway upregulates the levels of CD8^+^ T, IFN-1, and NK cells. Further, it leads to immune-system activation and the targeted killing of tumor cells, while glioma-cell growth is inhibited, together with tumor metastasis and invasion [66]. To summarize, circSOBP enhanced IFN-1 transcriptional activation by directly blocking TKFC (MDA5 inhibitor) binding to MDA5 while indirectly inhibiting the Warburg effect. The Warburg effect increased glucose uptake and fermentation to lactate [67] and was characteristic of metabolic changes in cancer cells. CircSOBP affected glioma development via activating relevant immune signal pathways [56].

Although circRNAs interact with IFN, other immune cells are involved in the tumor and cooperate with circRNAs. Considering that circNEIL3 is packaged into exosomes by hnRNPA2B1, leading to macrophage infiltration into the TME [22], this imbues circNEIL3 with the characteristic of immune evasion by stabilizing the oncoprotein IGF2BP3, which is associated with TAMs. By correlating circRNAs with immunology and elucidating the roles of multiple factors in tumor progression, it is anticipated that significant advancements in the combined treatment of glioma will be carried out in the future.

### 8.6. The Role of Exosome-Mediated CircRNAs

Most cells, such as mast, tumor, dendritic, and neuron-shaped glial cells, can generate extracellular vesicles (30 to 150 nm diameter)—exosomes [68,69,70], transport various biomolecules, and facilitate intercellular communication [71]. The blood–brain barrier (BBB) limits most drugs and therapeutic agents in gliomas [72]. However, experimental findings suggest that neutrophil-derived exosomes (NEs-Exos) penetrate the BBB and exhibit neutrophil-like chemotactic properties [73]. Doxorubicin (DOX) is used clinically to treat gliomas [74], and studies indicate that combining DOX with NEs-Exos increases therapeutic efficacy. Moreover, gliomas inhibit endothelial cell apoptosis via the secretion of exosomes, which construct a positive and suitable environment for tumor-cell propagation [69,75].

Exosomes act as natural vesicular carriers that mediate intercellular signaling by transporting proteins, circRNAs, and other bioactive molecules, which regulate pathophysiological processes in gliomas [76]. The exosomal packaging of circ-0012381 into microglia promotes glioma-cell proliferation and invasion by activating the CCL2/CCR2 signaling pathway and inhibiting phagocytosis [77]. Another study identified exosomal circNEIL3 as being overexpressed in glioma tissue, where it facilitates tumor progression by affecting tumor-associated macrophages (TAMs) [22]. circWDR62 is upregulated in gliomas and is associated with poor prognosis. It promotes tumor migration and invasion by sponging miR-370-3p to regulate MGMT (O^6^-methylguanine DNA methyltransferase) expression while also facilitating TMZ resistance by transferring exosomal circWDR62 from TMZ-resistant to TMZ-sensitive cells [78]. Exosomal circHIPK3 similarly increases TMZ resistance and promotes tumor-cell proliferation [79].

Exosomes thus act as a double-edged sword, contributing to glioma progression by transferring circRNAs that support tumor proliferation and invasion within the TME while also serving as potential therapeutic vectors capable of transporting drugs across the BBB. Recently, studies have reported that exosome production has made some achievements. Yang Z reported that a cellular-nano poration method produced large quantities of exosomes [80]. Nanocarrier-mediated drug delivery is increasingly becoming an effective and noninvasive method to deal with malignant glioma, which shows that exosomes have a decisive position in glioma therapy [81]. Future research may focus on optimizing exosomal circRNAs for targeted and precise glioma treatment.

## 9. The Clinical Relevance of circRNAs in Glioma

### 9.1. Diagnostic and Predictive Value of circRNAs

Pathological tissue biopsy remains the gold standard for cancer diagnosis. Over the past two decades, various studies have identified dysregulated circRNAs in glioma, prompting more detailed investigations into their regulatory pathways. Mechanistically, circRNAs affect glioma progression through epigenetic regulation, transcriptional and translational modulation, protein translocation, and immune cell recruitment. Their fluctuating expression levels and inherent stability during disease progression offer prognostic insight and correlate with glioma severity. For instance, circATIC is highly expressed in radioresistant glioma cells, promoting tumor progression by inhibiting miR-520d-5p expression. Downregulation of circATIC restores miR-520d-5p activity, therefore reversing tumor radioresistance [82]. Similarly, knockdown of circPITX1 [45] and circRYK [24]—both of which are overexpressed in glioma—has been shown to suppress tumor proliferation and migration. These findings suggest that dyregulated circRNAs with tumor-suppressive functions could serve as therapeutic targets, Increasing the expression of these inhibitory circRNAs may represent a novel therapeutic strategy, whereas upregulated circRNAs may serve as diagnostic or predictive biomarkers, Therefore, the diagnostic utility of circRNAs requires further attention. Combining circRNAs expression profiles with conventional tumor biomarkers could significantly improve the specificity and sensitivity of glioma diagnosis in the near future [83].

### 9.2. Roles of circRNAs in Resistance to Current Glioma Therapies

According to recent reviews, circRNAs contribute to glioma resistance primarily through two mechanisms: radiotherapy resistance and chemotherapy resistance. In terms of radiotherapy, as previously noted, circATIC plays a central role in mediating radioresistance. By acting as a competing endogenous RNA (ceRNA), circATIC sponges miR-520d-5p, reducing its inhibitory effects on Notch2 and Hey1, promoting tumor growth and invasion. Treatment with atomoxetine (ATX) downregulates circATIC, mitigating radioresistance [82]. Similarly, circATP8B4 sponges miR-766 [84], and circ-0008344 sponges miR-433-3p [85], both contributing to reduced radiosensitivity. Regarding chemotherapy, resistance to TMZ—the standard chemotherapeutic agent for glioma—remains a major clinical challenge [86,87,88]. CircASAP1 has been shown to promote TMZ resistance and glioma-cell proliferation. Its knockdown restores TMZ sensitivity by sponging miR-502-5p, leading to the dysregulation of NRAS (a Ras family member) and activation of MEK1/ERK1/2 signaling [89]. Similarly, miR-505-5p directly targets the 3′ UTR of AUF1, affecting glioma growth, proliferation, invasion, and angiogenesis [90]. The interaction between circRNAs and AUF1 remains an area of active investigation. Exosomal circRNAs are also implicated in chemoresistance. For example, exosomal circHIPK3 contributes to TMZ resistance by sponging miR-421 and upregulating zinc finger protein expression in the cerebellum [79]. Other exosomal circRNAs, such as circ-0042003 [91] and circ-0043949 [92], have similarly been shown to induce TMZ resistance.

### 9.3. Prospective Biological Mechanisms of Circrnas in the Development of Clinical Drugs

#### 9.3.1. The Cooperation Between CircRNAs and EGFR in Gliomas

**Figure 4:**
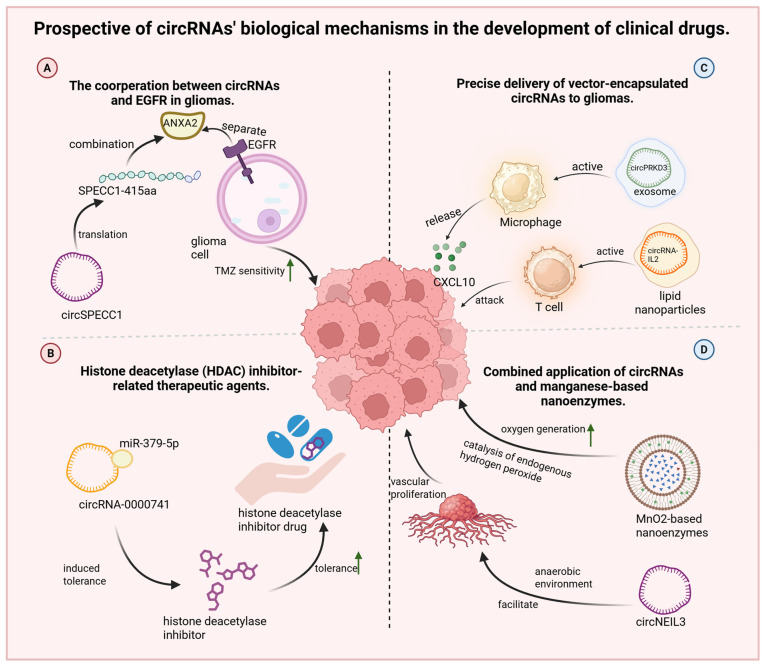
Prospective of circRNAs’ biological mechanisms in the development of clinical drugs. (**A**) CircSPECC1 can translate SPECC1 amino acid, which competitively binds annexin A2 protein, disrupting the binding of ANXA2 to EGFR and recovering the TMZ sensitivity of gliomas. (**B**) CircRNA-0000741, overexpressed in gliomas, can induce tolerance and impair HDAC inhibitor sensibility via sponging miR-379-5p. (**C**) Exosomemal circPRKD3 promotes tumor-associated macrophages secreting CXCL10; lipid nanoparticles loaded with circRNAs are inserted into the IL-2 coding sequence, which activates the proliferation of CD8+ T-cells and attracts cancer cells. (**D**) MnO_2_ nanoenzymes promote the generation of O_2_ from endogenous hydrogen peroxide (H_2_O_2_) in tumor tissues; circNEIL3 plays a role in promoting angiogenesis in tumor tissues in anaerobic environment. ↑, it represents the meaning of elevation and promotion.

The potential of circRNAs in clinical applications is increasingly recognized, particularly in relation to EGFR signaling. A recent study demonstrated that the protein SPECC1-415AA, encoded by circSPECC1, can competitively bind annexin A2 (ANXA2), disrupting the interaction between ANXA2 and EGFR. This interference plays a key role in restoring TMZ sensitivity in drug-resistant glioma cells (Figure 4A) [93]. The authors of this study are further investigating EGFR downstream pathways, specifically NF-κB and PI3K-AKT signaling cascades. Given the importance of EGFR signaling, particular attention should be paid to the *Ras* gene, a key downstream effector of EGFR. As observed in colorectal cancer [94], Ras mutations can lead to constitutive pathway activation that is independent of upstream EGFR autophosphorylation. Therefore, sequencing of the *Ras* gene should be performed when analyzing EGFR-related mechanisms in gliomas to rule out confounding effects from Ras mutations.

#### 9.3.2. Histone Deacetylase (HDAC) Inhibitor-Related Therapeutic Agents

Resistance to HDAC inhibitors remains a significant barrier to glioma treatment. However, HDAC-targeted agents have demonstrated favorable BBB permeability and antitumor activity in early-stage studies [95]. Liang et al. further identified circRNA-0000741 as a regulator of HDAC inhibitor (suberoylanilide hydroxamic acid, SAHA) tolerance in glioma cells [20]. In this context, circRNA-0000741 functions by sponging miR-379-5p, modulating key resistance pathways (Figure 4B). Theoretically, the downregulation of circRNA-0000741 could help restore HDAC inhibitor sensitivity. Importantly, several pathways for circRNA degradation have been elucidated [96], including RNase H-mediated cleavage [97], AGO2-dependent [98] and GW182-dependent [99] degradation, and m^6^A modification–mediated decay [100].

#### 9.3.3. Precise Delivery of Vector-Encapsulated CircRNAs to Gliomas

The presence of the BBB significantly limits drug efficacy, highlighting the urgent need for delivery systems that can achieve precise and efficient targeting. Recent findings show that exosome-encapsulated circPRKD3 promotes CXCL10 secretion by reprogramming tumor-associated macrophages, increasing CD8^+^ T cell recruitment and tumor infiltration. However, due to uncertainties regarding the safety of exosomes in humans, researchers have explored lipid nanoparticles (LNPs) as alternative carriers. These LNPs exhibited a similarly robust antitumor effect [101]. In another study, ursodeoxycholic acid-based lipid nanoparticles (ULNPs) were used to deliver circRNAs engineered to encode interleukin-2 (IL-2). This construct activated CD8^+^ T cell proliferation and improved the immune response against glioma cells. The formulation conferred improved half-life and a significant tumor-suppressive effect (Figure 4C) [18].

#### 9.3.4. Combined Application of CircRNAs and Manganese-Based Nanoenzymes

Emerging studies have revealed that circNEIL3 contributes to angiogenesis in glioma tissues [22], a process promoted by EWSR1, whose activity is elevated under hypoxic conditions. Consequently, we can envisage that therapeutic strategies to modify the tumor microenvironment could suppress angiogenesis and limit tumor progression. Increasing intratumoral oxygen levels, co-administering hydrogen peroxide and anti-angiogenic agents such as VEGF inhibitors, and using drug-retaining scaffolds like hydrogels [18] may collectively disrupt the tumor’s nutrient supply while minimizing systemic toxicity by restricting hydrogen-peroxide diffusion into healthy brain tissue. Interestingly, this conceptual framework aligns with recent advances involving manganese dioxide (MnO_2_)-based nanoenzymes. These materials catalyze the conversion of endogenous hydrogen peroxide into molecular oxygen (O_2_), enhancing oxygen-dependent tumor therapies under hypoxic conditions (Figure 4D). Manganese ions possess mild toxicity, while MnO_2_ nano- and microrobots have demonstrated effective tumor-penetration capabilities [16]. Manganese-based nanoenzymes, therefore, present considerable promise for improving both drug delivery and therapeutic efficacy in gliomas. However, despite their potential, no current studies have explored the combined application of manganese-based nanoenzymes and circRNAs in gliomas. Future research in this direction is warranted. Integrating these nanoenzymes with delivery vectors such as exosomes, lipid nanoparticles, or hydrogels may further improve intratumoral circRNA delivery and therapeutic outcomes.

Hyperbaric oxygen therapy may be another direction for glioma patients, and studies have shown that hyperbaric oxygen can indeed increase drug sensitivity in glioma patients [102]. Although the mechanism has not yet been fully elucidated, it is known that HIF-1 and HIF-2 are highly expressed in hypoxic environments. Once hyperbaric oxygen treatment facilitates cell proliferation and chemosensitivity, the expression of HIF1α and HIF2α is decreased. However, another piece of the literature demonstrated that the HIF-1 signaling pathway is closely associated with six types of circRNAs differentially expressed under hypoxia in gliomas. When inhibiting the expression level of circ-0000745, the experimental results can be clearly observed that the proliferation, migration, and invasiveness of tumor cells are inhibited [103]. In future studies, it will be of great necessity to elucidate the relationship between hyperbaric oxygen, HIF, and circRNAs, which also may be a promising direction for enhancing drug efficacy as well as restoring drug sensitivity.

## 10. Current Knowledge Gaps and Methodological Challenges in CircRNA Research in Gliomas

Although growing evidence suggests that circRNAs are involved in the pathophysiology of gliomas, significant gaps in knowledge and technical limitations persist, impeding clinical translation. First, the understanding of circRNAs remains fragmented. Key aspects such as their origin, biological function, degradation pathways, and the mechanisms governing their back-splicing events are not yet well-defined. Many current findings are derived mainly from bioinformatics predictions, with limited experimental validation. Robust, reproducible in vitro and in vivo studies are essential to confirm computational hypotheses and clarify their biological roles. Advances in RNA sequencing technologies are expected to facilitate these investigations and improve resolution in identifying functionally relevant circRNAs [104]. Second, the drivers behind circRNA dysregulation in gliomas remain poorly understood. Reports of differentially expressed circRNAs vary widely between studies, possibly due to inconsistencies in sample handling, preservation methods, experimental platforms, or patient staging. Given that gliomas are a dynamic and heterogeneous disease, circRNAs profiles may shift with disease progression, further complicating analysis. The standardization of experimental conditions and stratification of clinical samples are necessary to distinguish true biological signals from technical artifacts. Finally, the clinical safety and efficacy of circRNA-based therapeutics are still uncertain. Although preclinical studies suggest promising effects, comprehensive animal models are urgently needed to assess potential toxicities, off-target effects, and pharmacodynamics. Establishing robust safety profiles will be critical for accelerating the development of circRNA-targeted therapies and facilitating their translation into clinical trials. Ultimately, such advances can potentially improve outcomes and quality of life for patients with gliomas.

## 11. Future Research Directions of CircRNAs in Gliomas

To effectively regulate circRNAs, research should focus on elucidating their biogenesis, primary mechanisms, and degradation pathways. Several key areas require further investigation. First, since circRNAs are formed through the back-splicing of precursor mRNA exons, identifying regulatory elements that affect their production may allow the regulation of circRNA expression to control glioma progression. Second, recent studies indicate that circRNAs are involved in cellular energy metabolism, a process closely associated with succinylation. Given that both circRNAs and succinylation affect mitochondrial oxidative stress and metabolic regulation, exploring their specific interactions may reveal novel therapeutic targets. Third, circRNAs affect tumor development by binding to RBPs and miRNAs at specific sites, therefore activating downstream signaling cascades. Identifying these binding sites and designing high-affinity therapeutic molecules could facilitate the clinical application of circRNA-based treatments. Fourth, the development of circRNA-based vaccines represents a promising direction for glioma immunotherapy. CircRNAs exhibit higher structural stability than linear RNAs and can encode functional peptides. For example, the circRNA-derived protein SPECC1-415AA regulates EGFR and restores sensitivity to TMZ in glioma cells [93]. Similarly, circCOPA encodes COPA-99AA, a protein that inhibits glioma-cell proliferation, migration, and invasion by disrupting the NONO-SFPQ complex, which has a specific affinity for COPA-99AA [105]. Fifth, the refinement and development of delivery vectors capable of transporting circRNAs efficiently across the BBB is of significant importance. Due to the selective permeability of the BBB, many therapeutic agents fail to reach effective concentrations in CNS tissues. Innovative delivery systems that transport circRNAs for gene regulation or facilitate cytotoxic drug delivery at the cellular level could overcome this barrier. Future studies should focus on improving drug bioavailability, enhancing tumor-targeting specificity, and minimizing CNS toxicity, which remains a concern for carriers with high lipid solubility [95]. Recent findings highlight the use of hydrogel-based delivery systems, which demonstrate dual advantages: reducing systemic toxicity by lowering circulating drug concentrations and increasing localized drug accumulation within tumor tissues [18]. Continued exploration of such platforms, alongside exosomes and lipid nanoparticles, is expected to advance the development of circRNA-based interventions for gliomas.

## 12. Conclusions

This review discusses the multiple circRNAs implicated in the progression of glioma tumorigenesis. It also expands the scope of other molecules and mechanisms associated with the interaction with circRNAs, which are also the primary focus of clinical settings. CircRNAs are frequently dysregulated in gliomas and regulate tumor progression through several mechanisms. Beyond their pathological roles, circRNAs demonstrate significant promise in clinical diagnostics, prognostics, and therapeutic development. While various dysregulated circRNAs have been identified during glioma progression, and several of their pathogenic mechanisms have been elucidated, translating these findings into clinically applicable therapies remains limited. This gap is largely due to the current insufficiency in understanding the fundamental biology of circRNAs, as well as methodological limitations in studying their functions. Further research is needed to elucidate their regulatory roles, which may provide valuable insights for improving glioma prognosis and prolonging patient survival.

## Data Availability

The data that support the findings of this study are available from the corresponding author upon reasonable request.

## Supplementary Materials

The following supporting information can be downloaded at: https://www.mdpi.com/article/10.3390/biology14070795/s1, Table S1: The primary mechanism of circRNAs; Table S2: The role of circRNAs in glioma development and invasion. References [106,107] are cited in the Supplementary Materials.

## Abbreviations

aa	amino acids
ANXA2	annexin A2
BBB	blood–brain barrier
BTB	blood–tumor barrier
CNS	central nervous system
Circ-E-Cad	circular E-cadherin
CircRNAs	circular RNAs
ciRNAs	circular intronic RNA
CL	classical
DOX	doxorubicin
DMD	Duchenne muscular dystrophy
eIciRNAs	exon-intron circRNAs
ecircRNAs	exonic circRNAs
FGFR1	fibroblast growth factor receptor 1
GBM	glioblastoma
GECs	glioma endothelial cells
HDAC	Histone deacetylase
IFNs	interferons
ITGB8	integrin subunit beta 8
LNPs	lipid nanoparticles
miRNAs	microRNAs
MES	mesenchymal
M6A	N^6^-methyladenosine
MREs	microRNA response elements
MGMT	O^6^-methylguanine DNA methyltransferase
NEs-Exos	neutrophil-exosomes
PN	proneural
pre-mRNA	precursor mRNA
RBPs	RNA-binding proteins
SRSF3	serine/arginine-rich splicing factor 3
SRSF1	serine and arginine-rich splicing factor 1
TAMs	tumor-associated macrophages
TMZ	Temozolomide
TME	tumor microenvironment
tricRNAs	tRNA intronic circRNAs
ULNPs	ursodeoxycholic acid–based lipid nanoparticles
