# Supplementary material for: The Role of Circular RNA in the Progression of Gliomas and Its Potential Clinical Applications
Source: Biology (Basel). 2025 Jun 30;14(7):795. doi: 10.3390/biology14070795 (PMC12292135; doi:10.3390/biology14070795)
Supplement: Supplementary file 1 [file biology-14-00795-s001.zip › Table S1.docx]

| **Table S1. The primary mechanism of circRNAs.** | | | |
| --- | --- | --- | --- |
| The basic mechanism of circRNAs | Candidate circRNAs | Expression status in glioma | The influence in glioma tumorigenesis and proliferation. |
| Interact with RBPs | CircSMARCA5 | Down-regulate | The knockdown of circSMARCA5 disinhibite expression of RBP SRSF1 and promote splicing pattern of *SRSF3* mRNA, further regulates the expression of PTBP1 to prompt GBM cells migration. |
|  | CircRYK | Upregulate | promotes epithelial-mesenchymal transition in GBM and keep the stability of mRNA VLDLR by connecting with the RBP HuR. |
|  | CircATXN1 [1] | Upregulate | RBP SRSF10 combined with circ-ATXN1 promoted the generation of circ-ATXN1, and circ-ATXN1 enhanced cell viability and migration |
|  | CircNEIL3 | Upregulate | CircNEIL3 promotes its immunosuppressive properties and elevates its expression level coordinates with IGF2BP3 protein. |
| Ce-RNA mechanism | CircATIC | Upregulate | CircATIC mediating glioma radio-resistance and promoting tumor growth and invasion by sponges miR-520d-5p. |
|  | CircATP8B4 | Upregulate | CircATP8B4 reduce radiosensitivity by sponging miR-766. |
|  | CircHIPK3 | Upregulate | Exosomal circHIPK3 contributes to TMZ resistance by sponging miR-421. |
|  | CircSMARCA5 | Upregulate | Exosomal circSMARCA promote proliferation and migration of glioma by sponging miR-127-5p. |
|  | Circ-0088732 [2] | Upregulate | Circ-0088732 accelerated glioma progression through reducing miR-661 to increase RAB3D expression. |
|  | Circ-0008344 | Upregulate | Circ-0008344 down-regulation inhibited glioma growth and acted on miR-433-3p/RNF2 axis to enhance the radiosensitivity in glioma. |
|  | CircASAP1 | Upregulate | CircASAP1 can promote TMZ resistance and glioma cell proliferation by sponging miR-502-5p. |
| CircRNAs regulate transcription and affect their parental genes | CircSMARCA5 | Down-regulate | CircSMARCA5 downregulation upregulate SRSF1, which may promote the skipping of exon 4 in *SRSF3* pre-mRNA, further regulates the expression of PTBP1 that is positively regulate glioma cells migration. |
| Translation of circRNAs into functional proteins | CircAKT3 | Down-regulate | Circ-AKT3 encodes AKT3-174aa, AKT3-174aa compete with activated PDK1 and inhibits the proliferation, radiation resistance and tumorigenicity of GBM cells. |
|  | CircSPECC1 | Down-regulate | SPECC1-415AA encoded by circSPECC1, can competitively bind annexin A2, which can restore TMZ sensitivity in drug-resistant glioma cells. |
|  | CircFGFR1 | Down-regulate | Fibroblast growth factor receptor 1 is encoded by circFGFR1. |
|  | Circ-E-Cad | Upregulate | Circ-E-Cad encodes a secretory E-cadherin protein variant, which promotes GBM tumorigenesis and maintaining glioma stem cell tumorigenicity. |
|  | CircCOPA | Upregulate | CircCOPA encodes COPA-99AA, a protein that inhibits glioma cell proliferation, migration. |
| Interaction of circRNAs and viral mRNA in a pathological environment | CircRNP | Down-regulate | NF90/NF110 from circRNP complexes is released and binds with viral mRNA to improve antiviral immunity in viral infection. |
|  | Circ-0001400 | Upregulate | Circ-0001400 inhibit Kaposi's sarcoma-associated herpesvirus gene expression and promote TGF-α expression in KSHV. |
| Translocate proteins | CircAmotl1 | Upregulate | CircAmotl1 interacts with c-Myc, increasing its nuclear translocation, and increasing its affinity for target genes. |
|  | CircFOXP1 | Upregulate | CircFOXP1 promote the nuclear-to-cytoplasmic translocation of PTBP1 and increasing its expression in hepatocellular carcinoma. |
|  | CircBACH1 | Upregulate | CircBACH1 facilitating the translocation of HuR from the nucleus to the cytoplasm in hepatocellular carcinoma progression. |

**References**

1. Liu, X.; Shen, S.; Zhu, L.; Su, R.; Zheng, J.; Ruan, X.; Shao, L.; Wang, D.; Yang, C.; Liu, Y. SRSF10 inhibits biogenesis of circ-ATXN1 to regulate glioma angiogenesis via miR-526b-3p/MMP2 pathway. *J. Exp. Clin. Cancer Res. CR* **2020**, *39*, 121. https://doi.org/10.1186/s13046-020-01625-8.
2. Jin, T.; Liu, M.; Liu, Y.; Li, Y.; Xu, Z.; He, H.; Liu, J.; Zhang, Y.; Ke, Y. Lcn2-derived Circular RNA (hsa_circ_0088732) Inhibits Cell Apoptosis and Promotes EMT in Glioma via the miR-661/RAB3D Axis. *Front. Oncol.* **2020**, *10*, 170. https://doi.org/10.3389/fonc.2020.00170.
