# Supplementary material for: The Role of Circular RNA in the Progression of Gliomas and Its Potential Clinical Applications
Source: Biology (Basel). 2025 Jun 30;14(7):795. doi: 10.3390/biology14070795 (PMC12292135; doi:10.3390/biology14070795)
Supplement: Supplementary file 1 [file biology-14-00795-s001.zip › Table S2.docx]

| **Table S2. The role of circRNAs in glioma development and invasion** | | | | | |
| --- | --- | --- | --- | --- | --- |
| Functional validation | Candidate circRNAs | Expression status in glioma | Downstream targets | Mechanism and relevance of glioma | Clinical importance. |
| Angiogenesis and proliferation | CircNEIL3 | Upregulate | SPP1 protein | CircNEIL3 increases the protein expression of SPP1; EWS RNA-binding protein 1 increases the circularization of circNEIL3 | Potential prognostic biomarker and therapeutic target. |
|  | CircPITX1 | Upregulate | miR-584-5p | CircPITX1 mediates migration, invasion, and cell-cycle process by decreasing the level of miR-584-5p | Prospective biomarker and predicts value. |
|  | CircTTBK2 | Upregulate | miR-217 | Circ-TTBK2 decreases miR-217 expression and forms negative feedback in an Argonaute2-dependent manner to promote angiogenesis by CircTTBK2/ miR-217/ HNF1β/ Derlin-1 axis. | Promising predictive biomarker. |
|  | CircPTN | Upregulate | miR-145-5p and miR-330-5p | CircPTN rescued the inhibition of proliferation and downregulation of SOX9/ITGA5 by miR-145-5p and miR-330-5p. | Promising predictive biomarker and therapeutic target. |
|  | Circ-MAPK4 | Down-regulate | miR-125a-3p | Circ-MAPK4 was involved in facilitating p38/MAPK pathway by sponging and decreasing miR-125a-3p, which promotes glioma proliferation and apoptosis. | Prospective therapeutic target. |
|  | CircPOSTN | Upregulate | miR-361-5p | CircPOSTN targeted regulated TPX2 expression to impede apoptosis and proliferation via sponging miR-361-5p. | Potential therapeutic method for targeting glioma apoptosis. |
| Destroy normal cellular physiological processes | CircPTN | Upregulate | miR-145-5p | CircPTN promoted self-renewal and increased the expression of stemness markers (Nestin, CD133, SOX9, and SOX2) via sponging miR-145-5p. | Promising predictive biomarker: acts as an oncogenic factor. |
|  | CircTTBK2 | Upregulate | miR-761 | CircTTBK2 regulates ferroptosis by activating integrin subunit beta 8 (ITGB8) through the sponging of miR-761 | Promising predictive biomarker. |
| Disrupt cellular energetics | CircNFIX | Upregulate | miR-378e | CircNFIX protected RPN2 mRNA from degradation in glioma via sponging miR-378e, thus improving glucose metabolism | Potential prognostic biomarker and therapeutic target: inhibits apoptosis. |
|  | CircPOSTN | Upregulate | miR-361-5p | CircPOSTN targeted regulated TPX2 expression to impede proliferation and aerobic glycolysis via sponging miR-361-5p. | Potential therapeutic method for targeting glioma aerobic glycolysis metabolism. |
|  | CircSOBP | Down-regulate | TKFC proteins | CircSOBP inhibited glycolysis and activated the MDA5-mediated IKKε/TBK1/IRF3 signaling pathway by binding TKFC proteins. | Potential therapeutic method for targeting glioma metabolism. |
| Blood-brain barrier (BBB) and the blood-tumor barrier (BTB) | CircUSP1 | Upregulate | miR-194-5p | CircUSP1 preserved the barrier integrity and decreased its permeability, as well as raising tight junction-related protein claudin-5, occludin and ZO-1 expressions via sponging miR-194-5p. | Potential therapeutics strategy: regulates doxorubicin across BTB to induce apoptosis. |
|  | Circ-001160 | Upregulate | miR-195-5p | Circ-001160 promoted the expression levels of tight junction-related proteins by miR-195-5p/ ETV1 gene. | New targets for the treatment:  effectively promoted Dox through BTB. |
|  | CircDENND4 | Upregulate | miR-577 | CircDENND4 regulates BTB permeability and promotes the expression of miR-577 target genes ZO-1, occludin, and claudin-1 via through the cDENND4C/miR-577 axis | Chemotherapy therapeutic potential: enhanced doxorubicin delivery across BTB. |
| Mediate tumorigenesis by cytokines and immune cells | CircSOBP | Down-regulate | TKFC protein | CircSOBP upregulates the levels of CD8+ T, IFN-1, and NK cells via blocking TKFC binding to MDA5 and inhibiting the Warburg effect. | Potential therapeutic method immunological reprogramming. |
|  | CircNEIL3 | Upregulate | IGF2BP3 | CircNEIL3 leads to macrophage infiltration into the TME; mediates immune evasion by stabilizing the oncoprotein IGF2BP3. | Potential therapeutic target: immunotherapy. |
| Exosome-mediated circRNAs | Circ-0012381 | Upregulate | miR-340-5p | Exosomal circ-0012381 induced M2 microglia polarization by sponging miR-340-5p to suppressed phagocytosis and promoted the growth of the irradiated glioblastoma cells by CCL2/CCR2 axis. | Therapeutic potential: increases the efficacy of radiotherapy in glioma patients. |
|  | CircNEIL3 | Upregulate | IGF2BP3 | Exosomal circNEIL3 facilitates tumor progression by affecting tumor-associated macrophages via stabilizing IGF2BP3. | Potential prognostic biomarker and therapeutic target. |
|  | CircWDR62 | Upregulate | miR-370-3p | Exosomal circWDR62 facilitates TMZ resistance by transferring exosomal circWDR62 from TMZ-resistant to TMZ-sensitive cells by sponging miR-370-3p. | Promising therapeutic target and prognostic marker. |
